# *Lactobacillus plantarum*-Derived Inorganic Polyphosphate Regulates Immune Function via Inhibiting M1 Polarization and Resisting Oxidative Stress in Macrophages

**DOI:** 10.3390/antiox14040428

**Published:** 2025-04-01

**Authors:** Shuzhen Li, Aijuan Zheng, Zhimin Chen, Xiaoying Wang, Jiang Chen, Zhiheng Zou, Guohua Liu

**Affiliations:** 1 Key Laboratory for Feed Biotechnology of the Ministry of Agriculture and Rural Affairs, Institute of Feed Research, Chinese Academy of Agriculture Sciences, Beijing 100081, China; lishuzhen1543@163.com (S.L.); zhengaijuan@caas.cn (A.Z.); chenzhimin@caas.cn (Z.C.); wangxying313@163.com (X.W.); 2 Jiangxi Province Key Laboratory of Animal Green and Healthy Breeding, Institute of Animal Husbandry and Veterinary Science, Jiangxi Academy of Agricultural Sciences, Nanchang 330200, China; jianchen363@163.com (J.C.); zouzhihengxms@163.com (Z.Z.)

**Keywords:** inorganic polyphosphate, macrophages, oxidative stress, M1 polarization, immunomodulation

## Abstract

Inorganic polyphosphate (PolyP) is a high-molecular-weight polymer that plays multiple roles in regulating immune responses. However, the specific anti-inflammatory mechanisms of bacteria-derived PolyP are unclear. In the present study, PolyP was extracted from *Lactobacillus plantarum* (*L. plantarum*), and the chain length was estimated to be approximately 250 Pi residues. The immune regulatory functions of PolyP were investigated using a lipopolysaccharide (LPS)-induced RAW264.7 cell oxidative stress model, and dexamethasone was used as a positive control. The result revealed that both dexamethasone and PolyP were protective against oxidative stress by inhibiting macrophage M1 polarization and the production of several markers, such as nitric oxide (NO), reactive oxygen species (ROS), inducible nitric oxide synthase (iNOS), and cyclooxygenase (COX)-2. In addition, PolyP suppressed inflammation progression by regulating the production of several cytokines, such as interleukin (IL)-1β, interferon (INF)-γ, tumor necrosis factor (TNF)-α, and IL-6, and inhibited the expressions of *inhibitory κB kinase* (*IKK*) *α*, *IKKβ*, and extracellular regulated protein kinases 2 (*ERK2*). Conclusively, PolyP derived from *L. plantarum* has the ability to protect cells from oxidative stress damage by inhibiting M1 polarization in macrophages. These findings provide insights into the function of PolyP and offer support for the potential application of PolyP in immune-related diseases.

## 1. Introduction

PolyP is an immunologically active substance that can regulate immune responses in hosts. When targeting PolyP accumulation, the colonization, survival, and viability of bacteria in the host’s intestines would be affected, leading to the sensitization of pathogens to the chronic inflammatory environment [1]. Furthermore, PolyP may be a bioactive molecule produced by *Lactobacillus*, *Bifidobacterium*, and other intestinal flora that mediate the interaction between probiotics and their hosts and, therefore, promotes the performance of probiotic functions [2,3,4].

**Figure 1:**
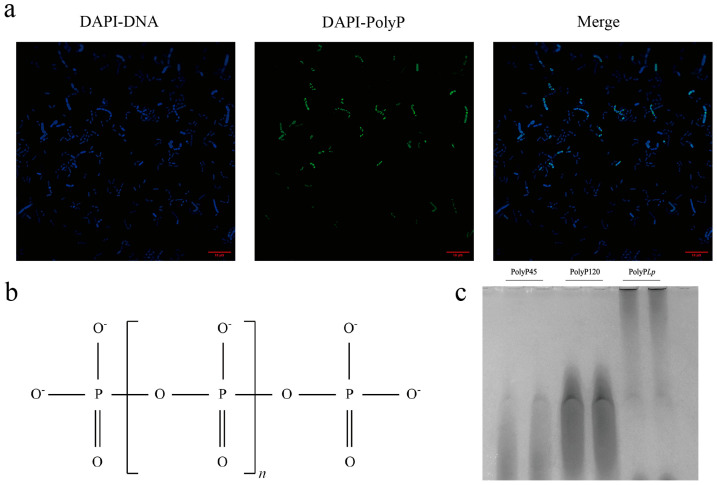
Structural characterization of inorganic polyphosphate (PolyP) derived from *Lactobacillus plantarum*. (**a**) PolyP granules in bacterial cells stained with DAPI (60× oil); (**b**) the structure of linearly condensed PolyP in bacteria; (**c**) the size distribution of PolyP on 8 M urea–PAGE gel (8%) and stained with *O*-toluidine blue; lanes1–2: molecular size marker (PolyP45), lanes 3–4: molecular size marker (PolyP120), and lanes 5–6: PolyP extracted from *Lactobacillus plantarum*.

As a linear polymer composed of several to hundreds of Pi residues linked by high-energy phosphorus anhydride bonds (Figure 1b), PolyP is widely found in microorganisms, plants, and animals and considered to be an ancient and universally conserved biomolecule [5,6,7]. PolyP can act as an active molecule both in vivo and in vitro and is thought to be promising for alleviating disease by mitigating inflammatory responses [8,9,10,11]. For example, PolyP produced by *Lactobacilli* is a bioactive molecule that induced cytoprotective HSP through the activation of the integrin–p38 MAPK pathway and prevented oxidant-induced intestinal barrier weakening [12]. Therefore, probiotic-derived PolyP may have the potential to enhance intestinal barrier functions and regulate intestinal disorders [13]. Inside the cell, PolyP has become a novel therapeutic target for the treatment of metabolic and acute or chronic inflammatory diseases [2]. For example, PolyP-kinase-defective *L. plantarum* strains significantly reduced the ability of Caco-2 cells to secrete the cytoprotective protein HSP27, suggesting that PolyP may be a key molecule that modulates hosts’ signaling pathways [14]. These findings provide new entry points for treating intestinal diseases with PolyP.

The inflammatory process is a complex biological response that occurs in immune cells, like macrophages [15]. Understanding the roles of inflammatory mediators is essential in comprehending the mechanisms underlying inflammation and developing effective anti-inflammatory therapies [16]. Macrophages are an important component of the innate immune system and play complex roles in chronic inflammatory disorders, metabolic processes, and inflammation [17]. Macrophages differentiate into classically activated macrophages (M1s) under stimulation and then release a variety of cytokines, such as NO, ROSs, TNF-α, and IL-6, to participate in various immune response pathways; eventually, the immune defense of macrophages is activated by an adaptive immune response. However, the persistence of the M1 phase or macrophage dysfunction can cause inflammatory disease and tissue damage, at which point macrophages polarize to an alternatively activated state (M2), and the formation of anti-inflammatory macrophages allows the activation of tissue repair and angiogenesis and has high-level scavenging activity that suppresses adaptive immune responses [18]. The functions of macrophages in health and disease have led to their consideration as potential targets for regulating immune diseases, thereby suggesting a promising strategy for maintaining the balance of hosts’ immune defenses and identifying regulators for managing inflammation and immune dysfunction diseases [19,20,21], especially in targeting the polarization of macrophages as a potential therapeutic intervention [22].

Long-chain PolyP is synthesized as a high-molecular-weight polymer in bacteria, and its effects on immunity might be highly conserved. However, exogenous PolyP has been demonstrated to have a complex relationship with innate and adaptive immune responses. However, the biological functions of PolyP remain unclear, including how it influences inflammation and hosts’ defense mechanisms and potentially impacts the process of infection in vitro. Prior to this, we used RAW 264.7 cells to establish immune stress models to clarify the immunomodulatory mechanisms of PolyP derived from *L. plantarum*.

## 2. Materials and Methods

### 2.1. DAPI Staining of PolyP in L. plantarum

The PolyP granules were stained with 2-(4-amidinophenyl)-6-indolecarbamidine dihydrochloride (DAPI) in living cells [23,24]. One milliliter of cells was centrifuged at 10,000× *g* for 15 min at 4 °C, and the cellular pellet was washed twice with 20 mM HEPES buffer (pH 7.5) before being resuspended in one milliliter of HEPES buffer. Then, 100 μM DAPI was added to a final concentration of 10 μM, and the mixture was incubated in the dark for 15 min at room temperature. Images were obtained via a DeltaVision ultrahigh-resolution microscope imaging system with a 60× oil objective lens (GE Healthcare Technologies Inc., Chicago, IL, USA). The fluorescence emitted by the DAPI-PolyP complex was observed at excitation/emission wavelengths of 415/550 nm, and the DAPI-DNA complex fluoresced at excitation/emission wavelengths of 360/450 nm.

### 2.2. Extraction and Purification of PolyP from L. plantarum

PolyP was isolated from *L. plantarum* with Glassmilk [25,26,27]. *L. plantarum* was cultured in MRS broth at 37 °C overnight, and 1 mL of each culture (OD600 = 1.5) was pelleted in a 1.5 mL sterile centrifuge tube. Then, the pellets were treated as follows: resuspension in 0.5 mL of guanidine isothiocyanate lysis buffer (GITC lysis buffer, 4 M guanidine isothiocyanate, 50 mM Tris-HCl, pH 7.0); incubation for 3–5 min at 95 °C; and sonication for 15 min. Then, 30 μL of 10% SDS, 0.5 mL of 95% ethanol, and 5 μL of Glassmilk (PD098, Omega, Bienne, Switzerland) were added to each tube, which was then centrifuged briefly to pellet the glass after mixing thoroughly. The supernatant was discarded, and the mixture was resuspended in 0.5 mL of new wash buffer (NW buffer, 5 mM Tris-HCl, 50 mM NaCl, 5 mM EDTA, 50% ethanol, pH 7.5). The mixture was then sonicated and centrifuged briefly, which was repeated twice. The washed pellet was then resuspended in 50 μL of nucleic acid removal buffer (50 mM Tris-HCl, 10 mM MgCl_2_, pH 7.4) and incubated at 37 °C for 1 h after adding DNase I and RNase A. The pellets were washed once with GITC lysis buffer and 95% ethanol, respectively, and then twice with NW buffer. PolyP was eluted from the pellet with 50 μL of 50 mM Tris-HCl (pH 8.0) at 95 °C for 2 min, after which the mixture was centrifuged, and the supernatant was collected. Finally, tubes equipped with a 3 kDa molecular-weight-cutoff (MWCO) membrane (Thermo Fisher Scientific, Waltham, MA, USA) were used to remove low-molecular-weight components, including ATP and short-chain PolyP, from the eluate, and the filtrate was vacuum concentrated (Eppendorf Concentrator Plus, Hamburg, Germany).

### 2.3. Quantification of Extracted PolyP

The Pi content of the extracted PolyP was determined according to already reported methods [24,28]. PolyP45 (S4379, Sigma-Aldrich, St. Louis, MO, USA) standards were prepared in sample buffer at final concentrations of 0.0 or 3.0 μmol/L Pi.

### 2.4. PolyP Chain Length Analysis Using Urea–PAGE

The extracted PolyP was separated using urea–PAGE to determine the chain length [27,29,30]. The gel mixture consisted of 8 M urea, 8% PAGE (acrylamide:bisacrylamide = 29:1), 5× TBE buffer (450 mM Tris, 450 mM borate, and 13.5 mM EDTA, pH 8.3), 0.5% ammonium persulfate (10% *w*/*v*), and 0.05% TEMED (Amresco, Washington, DC, USA), and the gels were pre-electrophoresed at 150 V for 0.5 h. PolyP (2 mg/mL) was mixed with 5× loading buffer (5× TBE buffer, 50% sucrose, and 0.125% bromophenol blue, pH 8.3). Electrophoresis was performed at 150 V for 1 h with 1× TBE buffer. PolyP was detected by staining for at least 1 h with *O*-toluidine blue (0.05%) in 25% methanol and 5% glycerol and destaining for 2–3 h in staining buffer without *O*-toluidine blue.

### 2.5. Cellular Culture and Treatments

The murine macrophage cell line RAW 264.7 (ECACC, Salisbury, UK) was cultured in DMEM, with a glucose concentration of 1 g/L, supplemented with 10% FBS at 37 °C, 5% CO_2_, and 95% humidity. Prior to the establishment of LPS-induced immune responses (L4391, Sigma-Aldrich), the cells were pretreated for 2 h with PolyP or dexamethasone (DEX) (HY-14648G, MCE, Monmouth Junction, NJ, USA), followed by coincubation for 24 h.

### 2.6. Cellular Proliferation Assays and Morphological Observations

A cell-counting kit-8 (C0038, Beyotime Biotech Inc., Shanghai, China) was used to evaluate cellular viability. RAW 264.7 cells were split at a density of 50 000 cells per well. The CCK-8 reagent was added to each well after treatment, and the samples were incubated for 1 h. Absorbance at 450 nm was measured via a microplate reader (BioTek Synergy H1, Agilent Technologies Inc., Santa Clara, CA, USA).

Live cellular imaging was performed with a ZenCELL owl incubator microscope to analyze the dynamic changes in and developments of RAW264.7 cells. The RAW264.7 cell line was seeded in 24-well plates at a density of 1.5 × 10^5^ cells/well, and each treatment had 4 replicate wells, inserted in the ZenCELL owl incubator and grown for around 24 h. The incubation environment was maintained at 37 °C, 5% CO_2_, and 95% humidity. Pictures were taken in intervals of 1 h within 24 h.

The cellular morphology of the RAW 264.7 cells was captured with a ZEISS Axio Vert.A1 inverted microscope.

### 2.7. Nitric Oxide Estimation Using the Griess Reagent

The intracellular NO release of the RAW 264.7 cells was detected using the NO-assay-kit-based Griess method (S0021S, Beyotime Biotech Inc.). Cells were inoculated in 96-well plates at 1 × 10^4^ cells per well, and after 24 h of treatment, 50 μL of the cellular supernatant was collected from each well in different treatment groups and transferred to a new 96-well plate. The Griess reagent was added to determine the absorbance at 540 nm via a microplate reader (Agilent Technologies).

### 2.8. Reactive Oxygen Species Assay in Macrophages

The intracellular ROSs in the RAW 264.7 cells were detected via a DCFH-DA probe (HY-D0940, MCE). The cells were seeded in 6-well plates at a density of 2 × 10^5^/well, washed twice with PBS, and collected in EP tubes at the end of the treatment. Then, 1 mL of fresh DMEM containing H2DCFDA (10 μM) was added to each tube, and the mixture was incubated at 37 °C for 30 min in the dark. The pellets were resuspended in PBS after being washed twice with PBS, and the number of cells in the suspension was counted. Finally, the cellular suspension was transferred to a 96-well white plate, and the fluorescence for ROSs was recorded at 480/525 nm excitation and emission wavelengths with a plate reader (Agilent Technologies). The fluorescence value was normalized to the number of cells, and the results are expressed as the treatment group versus the control group.

### 2.9. ELISAs

ELISA kits for cytokines were purchased from KIRbio (JINZHIYAN Biotechnology, Beijing, China). The cytokine levels in the cell-free supernatants were analyzed according to the manufacturer’s instructions.

### 2.10. Total RNA Extraction and qRT-PCR

The total RNA in the cells was isolated using the RNA Easy Fast Tissue/Cell Kit (DP451, Qiagen, Beijing, China) and then reverse transcribed with the EasyScript^®^ One-Step gDNA Removal and cDNA Synthesis SuperMix (AE311-02, TransGen Biotech, Beijing, China). Quantitative PCR was performed using a C1000 Touch^TM^ with a CFX real-time PCR detection system (Bio-Rad Laboratories, Hercules, CA, USA) and PerfectStart^®^ Green qPCR SuperMix (AQ601-01-V2, TransGen Biotech). Target gene expressions were compared between samples by normalization to GAPDH expressions and applying the 2^−ΔΔCt^ formula. The primer sequences are listed in Table 1.

### 2.11. Statistical Analyses

Statistical analyses were carried out using SPSS 24, and the significance was calculated using one-way ANOVA and Duncan’s multiple comparison test. All the data for the experiments are presented as the means ± S.D.s of at least three data points from three different experiments (* *p* < 0.05, ** *p* < 0.01, and *** *p* < 0.001).

## 3. Results

### 3.1. PolyP Granule Accumulation in L. plantarum

PolyP is a polymer formed by high-energy phosphoanhydride bonds connecting orthophosphate groups (Figure 1b). The chemical structure of PolyP is similar to that of DNA in that both contain strands of orthophosphate groups, and DAPI is permeable to DNA, so it may bind to PolyP particles. When the excitation wavelength was 415 nm, yellow–green fluorescence was detected at an emission wavelength of 550 nm for DAPI-PolyP, while blue fluorescence was detected at excitation/emission wavelengths of 360/450 nm for the DAPI-DNA complex. The overlap in the merged figure indicates that the PolyP was bound to the nucleic acid (Figure 1a).

### 3.2. The Quantification and Chain Length of PolyP Derived from L. plantarum

In this study, PolyP45 was used as a standard to establish a standard curve of concentration versus fluorescence values, Y = 31950 × X + 2313, R^2^ = 0.9989. The Pi content of the extracted PolyP (1 mg/mL) was calculated, from the standard curve, at 1.30 μmol/L Pi.

The polymer lengths of PolyP were estimated using 8% urea–PAGE gels and commercially available PolyP45 and PolyP120 ladders. PolyP with three different chain lengths migrated and was separated via PAGE, resulting in different mobilities: PolyP45 (lanes 1–2), PolyP120 (lanes 3–4), and PolyP derived from *L. plantarum* (5–6) (Figure 1c). Finally, compared with the PolyP ladder, the PolyP ladder extracted from the strain presented a migration distance shorter than 120 Pi units; thus, the average chain length of the PolyP was estimated to be approximately 250 Pi residues.

### 3.3. Oxidative Stress Model Establishment

**Figure 2:**
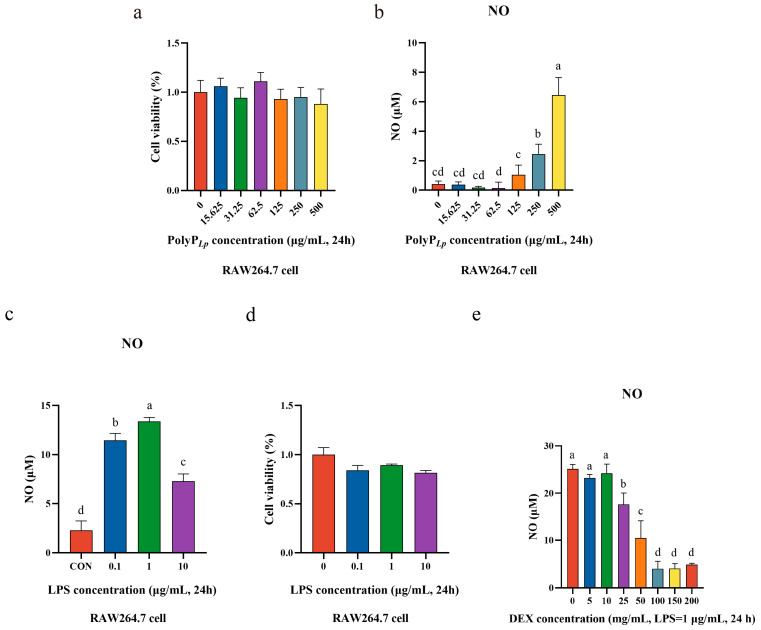
Immune stress model establishment. (**a**) Cellular viabilities of RAW264.7 cells at different PolyP treatment concentrations; (**b**) NO production after treatment with different concentrations of PolyP; (**c**) NO production of RAW264.7 cells stimulated using LPS; (**d**) cellular viabilities of RAW264.7 cells at different LPS treatment concentrations; (**e**) NO production after treatment with different concentrations of DEX. Bars with different superscripts (**a**–**d**) represent statistically significant differences (*p* < 0.05).

We determined the effect of the PolyP concentration on the cellular viability using the CCK8 method and determined the optimal treatment concentration. The results revealed that different concentrations of PolyP had no inhibitory effect on the cellular viability, and a concentration of 62.5 μg/mL significantly promoted cellular viability (Figure 2a); in addition, low concentrations of PolyP did not stimulate oxidative stress in cells, whereas higher concentrations of PolyP stimulated the production of NO in cells (Figure 2b). Therefore, 62.5 μg/mL concentrations were selected for performing all the experiments to explore the immune-regulatory function of PolyP for RAW 264.7 cells.

In this study, we used LPS to stimulate RAW264.7 cells to establish an oxidative stress model. LPS stimulation induces oxidative stress in cells, which leads to the production of large amounts of oxidation products, such as NO and ROSs, and we evaluated the inflammation model by detecting the level of NO released by macrophages. It was found that LPS significantly stimulated the production of NO in cells, and the release of NO reached its highest level at a concentration of 1.0 μg/mL (Figure 2c); in addition, the cellular viability was above 80% for different concentrations of LPS, so an LPS concentration of 1.0 μg/mL and a stimulation time of 24 h were finally selected as the conditions for inducing the inflammation model for the subsequent experiments.

DEX, a steroidal drug used clinically in the treatment of many inflammatory diseases, can effectively inhibit LPS-induced inflammatory responses. Therefore, DEX was utilized as a positive control to compare the anti-inflammatory capacities of the PolyP in this experiment with those of PolyP derived from other sources. As the results show (Figure 2e), the level of NO in LPS-stimulated RAW264.7 cells gradually decreased with increasing DEX concentration, and the NO level no longer decreased at concentrations of 100 mg/mL and higher, so a DEX concentration of 100 mg/mL was selected for the subsequent experiments.

### 3.4. PolyP Has No Inhibitory Effect on Cellular Proliferation

**Figure 3:**
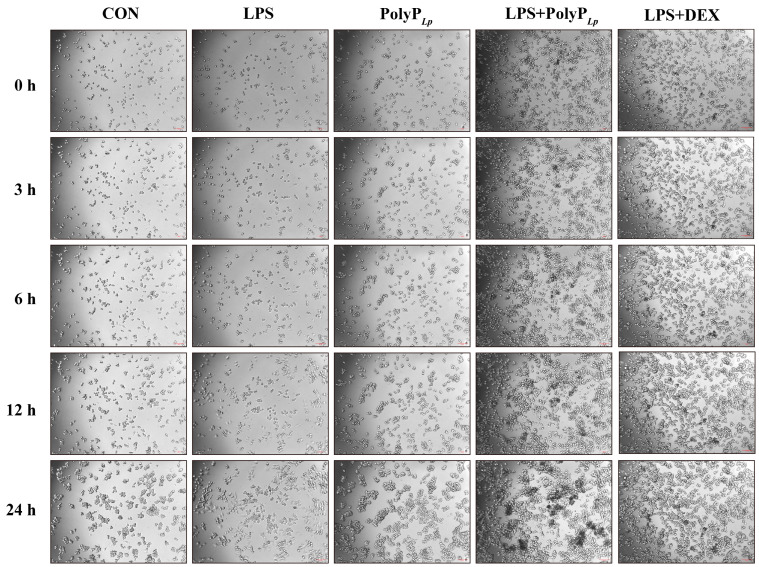
Effect of PolyP*_Lp_* on the proliferation of RAW 264.7 cells (10×, Scale bar: 200 μm).

As Figure 3 shown, the dynamic monitoring of the macrophages revealed that LPS stimulation affected macrophage proliferation, the cells were clearly differentiated by 12 h of stimulation, and the macrophages fully polarized to the M1 type and proliferated slowly during 12–24 h. In contrast, PolyP had no effect on cellular proliferation and morphology, and when PolyP acted on LPS-stimulated macrophages, the differentiation of the cells was suppressed and the proliferation tended to be normalized; these effects were the same as those obtained using DEX.

### 3.5. PolyP Repairs the Morphology of LPS-Activated Macrophages

**Figure 4:**
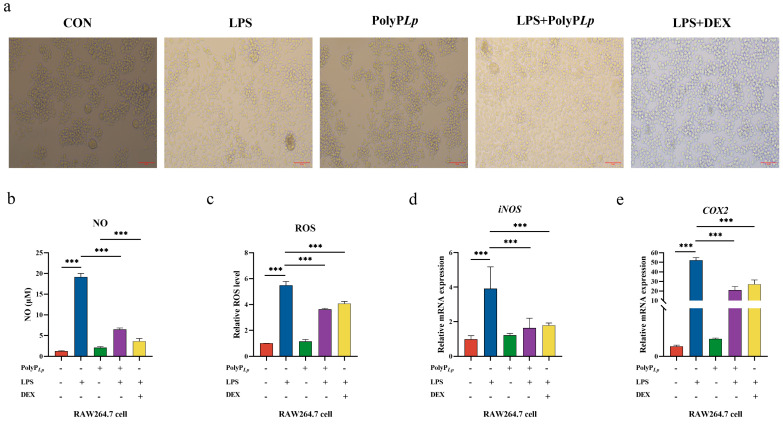
Effect of PolyP*_Lp_* on oxidative stress in RAW 264.7 cells induced by LPS. (**a**) The morphological changes of the cells (200×); (**b**) NO production; (**c**) ROS production, fluorescence values were normalized to the cellular number; (**d**) *iNOS* mRNA expression; (**e**) *COX2* mRNA expression. The data are presented as means ± S.D.s (*n* = 3). Differences were considered as significant at *** *p* < 0.001 compared with the control or model group (LPS).

Nonactivated macrophages showed a round or oval morphology with short protrusions, but the cells became flattened, and most of them were elongated or had pseudopods after stimulation with LPS, resulting in a strong anchorage dependency. In addition, even these polarized cells showed irreparable cytoplasmic leakage. However, PolyP treatment avoided the macrophage polarization caused by the LPS stimulation, and the number of cells with pseudopods or stretched shapes was significantly reduced. Similarly, DEX completely resisted the changes in the cellular morphology induced by LPS stimulation, allowing the cells to grow normally (Figure 4a).

### 3.6. PolyP Resists LPS-Induced Oxidative Stress in Macrophages

Oxidative stress occurs when intracellular oxidative and antioxidant effects are in a state of imbalance. Cells are predisposed to oxidative reactions, and the process usually produces a large number of oxidative intermediates. In macrophages, the ROS level is significantly increased under LPS activation, thus causing severe oxidative stress and cellular damage. LPS stimulation drastically increased the ROS level, promoted the release of NO, and increased the expressions of *iNOS* and *COX2*. Although PolyP decreased the ROS level, alleviated oxidative stress in LPS-induced RAW264.7 cells by decreasing the amount of NO released, and inhibited *iNOS* and *COX2* expressions, the effect was equivalent to that obtained using DEX (Figure 4b–e).

### 3.7. PolyP Regulates the Immune Response in LPS-Induced Macrophages at the Protein Level

**Figure 5:**
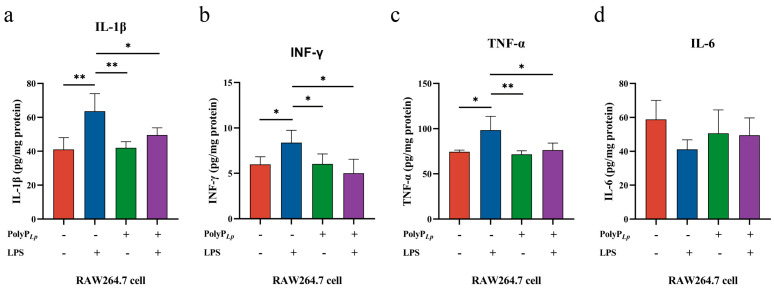
Effects of PolyP*_Lp_* on protein secretion of cytokines in RAW 264.7 cells: (**a**) IL-1β, (**b**) INF-γ, (**c**) TNF-α, and (**d**) IL-6. The data are represented as means ± S.D.s (*n* = 3). Differences were considered as significant at * *p* < 0.05 and ** *p* < 0.01 compared with the control or model group (LPS).

Under the continuous activation of LPS, immunocompromised macrophages secreted large amounts of inflammatory cytokines, such as IL-1β, INF-γ, TNF-α, and IL-6, while PolyP controlled the continuous aberrant inflammatory responses of activated macrophages by suppressing the levels of IL-1β, INF-γ, and TNF-α (Figure 5).

### 3.8. PolyP Regulates the Immune Response in LPS-Induced Macrophages at the mRNA Level

**Figure 6:**
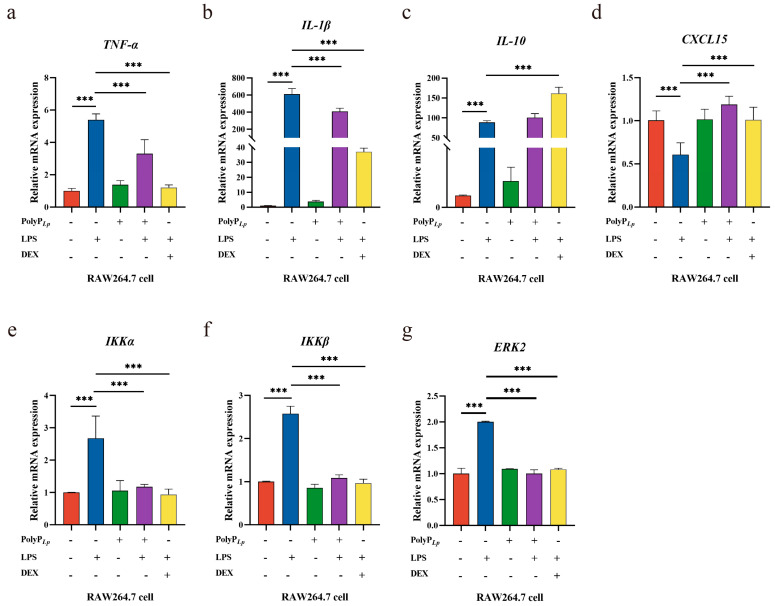
Effects of PolyP*_Lp_* on the mRNA expressions of cytokines in RAW 264.7 cells: (**a**) *TNF-α*, (**b**) *IL-1β*, (**c**) *IL-10*, (**d**) *CXCL15*, (**e**) *IKKα*, (**f**) *IKKβ*, and (**g**) *ERK2*. The data are represented as means ± S.D.s (*n* = 3). Differences were considered as significant at *** *p* < 0.001 compared with the control or model group (LPS).

In this experiment, LPS was used to stimulate macrophages to undergo a significant inflammatory response and induce M1 polarization, so we assayed the expressions of the key cytokines to explore the anti-inflammatory mechanism of the PolyP. LPS stimulated the expressions of the inflammatory mediators *TNF-α*, *IL-1β*, and *CXCL15*, while PolyP exerted anti-inflammatory effects by modulating the expressions of inflammatory mediators (Figure 6a–e). In addition, PolyP significantly reduced the expressions of *IKKα*, *IKKβ*, and *ERK2* in LPS-stimulated macrophages, and the regulatory effect was comparable to that obtained using DEX.

## 4. Discussion

### 4.1. The Chain Length of PolyP Determines Its Immunomodulatory Functions

PolyP, in bacteria, is predominantly distributed in the cytoplasm, with smaller amounts in organelles, such as the nucleus, cytoplasm, and mitochondria, and at the periphery of the cell [7,31]. Long-chain PolyP synthesized in bacteria easily binds to biomolecules, such as intracellular proteins, nucleic acids, lipids, and sugars, and exists in the form of particles, which are highly biologically active and are involved mainly in regulating the physiological and metabolic activities of organisms [7,31,32]. PolyP is synthesized at required lengths to perform different functions in the cell, while the effects of exogenous PolyP are chain length and dose dependent and are not highly correlated with its origin [3,33]. Long-chain PolyP possesses an anti-inflammatory function both in vitro and in vivo and can protect cells or hosts from stimulation and even repair damage safely and effectively [11,12,32]. However, short-chain PolyP has the opposite effect and may act as a mediator of pro-inflammatory responses in cells or by enhancing signaling, ultimately amplifying the inflammatory responses of immune cells and exacerbating inflammatory diseases [3,34]. In our study, PolyP was extracted from *L. plantarum* and purified to determine the chain length, which was approximately 250 Pi residues. According to the classification of PolyP chain lengths (shorter polymers have < 150 Pi residues and longer polymers have > 150 Pi residues), our PolyP belongs to the long-chain category, so it is likely to have immunomodulatory and anti-inflammatory functions [33,35].

### 4.2. PolyP Has No Toxic Effect on Macrophages

Macrophages have the exquisite ability to sense and respond to dynamically changing signals in the microenvironment, and feedback to stimuli can be clearly manifested in morphology; therefore, we not only investigated the effect of PolyP on cellular viability but also observed its effect on the cellular morphology. It was found that PolyP neither inhibited the proliferation of macrophages nor stimulated cellular polarization, and there was no oxidative stress stimulation of the cells, which indicated that PolyP had no toxic effect on macrophages and that the study of its role in immunomodulation could be continued. In contrast, the viability of LPS-stimulated cells was disturbed, and a significant oxidative stress response was stimulated, which subsequently induced a significant inflammatory response in the macrophages. When PolyP acted on LPS-treated macrophages, the cellular viability and morphology were not significantly different from those of normal cells, suggesting that PolyP may have a positive effect on macrophage regulation, which warrants further analysis and study.

### 4.3. PolyP Regulates the Immune Response by Targeting the Differentiation of M1 Macrophages

The morphology of macrophages reflects the immune status of cells. M1 macrophages are activated by cytokines, such as LPS, TNF-α, and IFN-γ, and then generate appropriate immune responses to participate in pathogen clearance and antitumor activities [36]. However, sustained exposure to inflammatory factors may potentiate the destructive inflammatory capability of M1 macrophages and, thus, exacerbate inflammatory processes that may be harmful to health [18]. Our results revealed that the macrophages differentiated into M1 macrophages that appeared round or flat after LPS stimulation. However, the morphology of the macrophages treated with PolyP was not significantly different from that of the LPS-treated cells, and the cellular morphology was complete and normal. Therefore, we think that PolyP can prevent LPS-induced macrophage polarization and protect cells from damage.

In the present study, long-chain PolyP maybe inhibit the polarization of macrophages by decreasing the production and release of M1 macrophage markers (iNOS, TNF-α, and IL-6). Previous studies have confirmed that exogenous long-chain PolyP (containing ~150 Pi residues) can help to protect the host against macrophage target recruitment as well as inflammation by suppressing the activation of the TNF-α-JNK/p38 pathway, thereby counteracting LPS-induced tissue damage and death [37]. In addition, treating mammalian immune cells with PolyP possessing different chain lengths (e.g., PolyP containing ~14, 60, and 130 Pi residues) revealed that PolyP acted as a regulator of innate immunity to inhibit the expression of iNOS induced by LPS in mouse macrophages and that the effect of the long-chain PolyP was the most significant [38].

### 4.4. PolyP Resists LPS-Activated Oxidative Stress in Macrophages

Oxidative stress in macrophages mediates inflammatory responses and cellular damage in hosts, and the modulation of this process has been implicated in a variety of diseases, such as acute lung injury, asthma, and other lung diseases; atherosclerosis; and metabolic disorders, such as obesity and diabetes, making it a target for intervention or the treatment of immune-related diseases [39,40,41]. The macrophage’s immune response is a complex and dynamic process involving various metabolic and signaling pathways and has been the focus of research on immunology and inflammation [42]. In this study, PolyP regulated the inflammatory response triggered by LPS by directly reducing the secretions or mRNA expressions of pro-inflammatory factors, such as IL-1β, TNF-α, ILL-6, and CXCL15, and by increasing the level and expression of IL-10 to mitigate the inflammatory damage to cells. High levels of ROSs induce oxidative stress and are associated with the production and release of inflammatory mediators, and excessive ROSs cause irreversible damage to cells. NO is a gaseous signaling molecule produced by iNOS through the depletion of L-arginine, both of which are rarely produced in healthy states but are only produced in large quantities when cells are stimulated by oxidative stress [43], and abnormal levels of NO are often caused by the dysregulated expression of iNOS during the development of immune disease [44,45]. COX2 is a key inducible enzyme in the process of prostaglandin biosynthesis and has very low activity in normal tissues and cells, but the expression of COX2 increases sharply once the cells are stimulated, which causes an increase in enzymatic products at the site of the inflammation and, ultimately, leads to an inflammatory response and tissue damage [46,47]. In our study, PolyP alleviated LPS-induced oxidative stress damage in macrophages by decreasing the expressions of iNOS and COX2 and, accordingly, reducing NO and ROS production in LPS-induced macrophages. On the other hand, the reduced expression of COX2 also facilitates the reprogramming of pro-inflammatory M1 macrophages to anti-inflammatory M2 macrophages, thereby alleviating the inflammatory response [48]. The above results demonstrate that long-chain PolyP protected against immune dysfunction and tissue damage [38,39]. In view of the abovementioned findings, we suggest that PolyP has the potential to be used as an anti-inflammatory agent, but more research models are needed to prove this hypothesis.

### 4.5. Superficial Mechanism of the Anti-Inflammatory Properties of PolyP

In the present study, large amounts of pro-inflammatory factors, such as IL-1β, TNF-α, and IL-6, were secreted after LPS stimulation, and cellular polarization synergizes with cytokines to enhance the effect of the LPS on inflammation, while PolyP can alleviate the inflammatory response by regulating the expressions of inflammatory factors, thereby reducing the damage, caused by inflammation, to macrophages. We detected the expressions of the signaling molecules of the NF-κB and MAPK inflammatory pathways associated with M1 polarization and found that PolyP inhibited IKKα, IKKβ, and ERK2 expressions, with the same effect as that obtained using DEX. IKKα and IKKβ are the two major IκB kinases and can activate NF-κB in response to various stimuli [49]. The MAPK cascade is a key signaling pathway that regulates various cellular processes, such as cellular proliferation, differentiation, apoptosis, and stress responses. In addition, the ERK/MAPK signaling pathway plays crucial roles in the survival and development of tumor cells [50,51]. In our study, both PolyP and DEX inhibited the activations of IKKα, IKKβ, and ERK2, which is important for anti-inflammatory drugs to inhibit the activations of the NF-κB and MAPK signaling pathways in LPS-stimulated macrophages. This result strongly suggests that PolyP has anti-inflammatory potential, but more research is needed to prove it.

## 5. Conclusions

**Figure 7:**
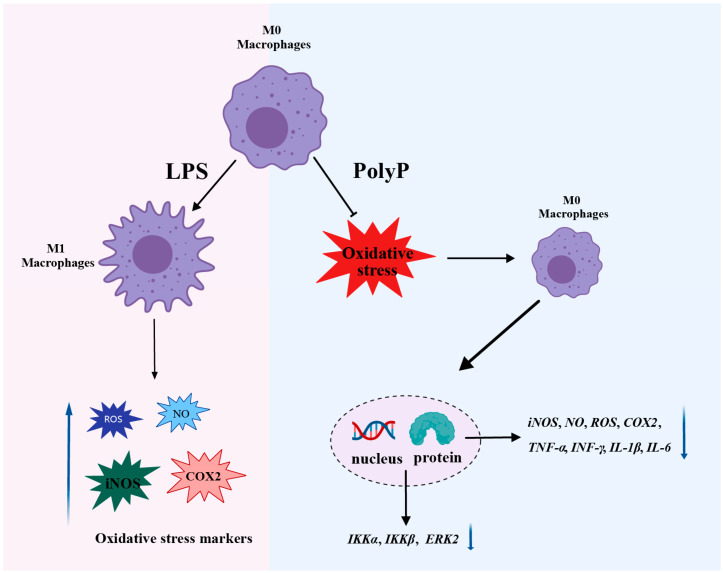
Schematic of the roles of PolyP in resisting oxidative stress and suppressing inflammation in LPS-induced RAW264.7 cells. Black arrows highlight findings from this study; T-arrow indicates that the process is inhibited; blue arrows indicate a change in level (increase or decrease).

The PolyP in this study was derived from *L. plantarum*, and the molecular weight was determined to be approximately 250 Pi residues. The ability of the PolyP to suppress inflammation progression was investigated via morphological- and molecular-level analyses. In summary, PolyP involves interfering with M1 polarization and resists oxidative stress induced by LPS in macrophages, thereby regulating the immune responses of macrophages (Figure 7). These findings not only provide insights into the functions of PolyP but also provide a basis for future research and potential applications of PolyP as a safe agent to promote immune functions. Our current study has revealed the immunomodulatory function of PolyP, and further studies are needed to investigate its mechanism of action in more disease models.

## Figures and Tables

**Table 1:** Primer sequences for RT-qPCR.

Gene Name	Primer Sequence
*IL-1β*	Forward: GCAGCAGCACATCAACAAGAGC Reverse: AGGTCCACGGGAAAGACACAGG
*IL-10*	Forward: TGTCATCGATTTCTCCCCTGTG Reverse: TTCATGGCCTTGTAGACACC
*iNOS*	Forward: TCTGCTGGCTTCCTGCTCTCC Reverse: TCTCCGTGGGCGTGTGATCC
*TNF-α*	Forward: GTGCCAGCCGATGGGTTGTAC Reverse: TGACGGCAGAGAGGAGGTTGAC
*COX2*	Forward: GACAGATTGCTGGCCGGGTTG Reverse: CAGGGAGAAGCGTTTGCGGTAC
*CXCL15*	Forward: TGGGTGAAGGCTACTGTTGG Reverse: AGCTTCATTGCCGGTGGAAA
*IKKα*	Forward: GCAGACCGTGAACATCCTCT Reverse: TCCAGGACAGTGAACGAGTG
*IKKβ*	Forward: AGGCGACACGTGAACAGAT Reverse: CTAAGAGCGGATGCGATG
*ERK*	Forward: GCAGATCCAGATCATGATCACAC Reverse: CTGTGACTGAAGATGGTGACTC
*GADPH*	Forward: GTAACCCGTTGAACCCCATT Reverse: CCATCCAATCGGTAGTAGCG

## Data Availability

The data that support the findings of this study are available from the corresponding author upon reasonable request.

## Abbreviations

PolyP	Inorganic polyphosphate
LPS	Lipopolysaccharide
DEX	Dexamethasone
NO	Nitric oxide
ROS	Reactive oxygen species
iNOS	Inducible nitric oxide synthase
IL-1β	Interleukin-1β
INF-γ	Interferon-γ
TNF-α	Tumor necrosis factor-α
IL-6	Interleukin-6
COX-2	Cyclooxygenase-2
DAPI	4′,6-Diamidino-2-phenylindole dihydrochloride
PAGE	Polyacrylamide gel electrophoresis
NF-κB	Nuclear factor kappa-B
MAPK	Mitogen-activated protein kinase
ERK	Extracellular regulated protein kinase
IKK	IκB kinase
CXCL15	C-X-C motif chemokine ligand 15
